# Crystal structure and Hirshfeld surface analysis of 1,2-bis­(2′,6′-diisoprop­oxy-[2,3′-bipyridin]-6-yl)benzene

**DOI:** 10.1107/S2056989018013002

**Published:** 2018-09-25

**Authors:** Ki-Min Park, Suk-Hee Moon, Youngjin Kang

**Affiliations:** a Research Institute of Natural Science, Gyeongsang National University, Jinju, 52828, Republic of Korea; b Department of Food and Nutrition, Kyungnam College of Information and Technology, Busan 47011, Republic of Korea; c Divisionof Science Education, Kangwon National University, Chuncheon 24341, Republic of Korea

**Keywords:** crystal structure, dipyridyl derivative, isoprop­oxy substituent, helical structure, Hirshfeld surface analysis

## Abstract

The title mol­ecule, C_38_H_42_N_4_O_4_, displays a helical structure induced by the combination of the C—C—C—C torsion angle [−10.8 (2)°] between two 2,3′-bipyridyl units attached to the 1,2-positions of the central benzene ring and consecutive connections between five aromatic rings through the *meta*- and *ortho*-positions. Intra­molecular C—H⋯π inter­actions between an H atom of a pyridine ring and the centroid of a another pyridine ring contributes to the stabilization of the helical structure. In the crystal, weak C—H⋯π inter­actions link the title mol­ecules into a two-dimensional supra­molecular network extending parallel to the *ac* plane, in which the mol­ecules with right- and left-handed helical structures are alternately arranged. Hirshfeld surface analysis and two-dimensional fingerprint plots indicate that the mol­ecular packing is dominated by van der Waals inter­actions between neighbouring H atoms, as well as by C—H⋯π inter­actions. One isopropoxyl group is disordered over two sets of sites [occupancy ratio 0.715 (5):0.285 (5)].

## Abstract 2

The title mol­ecule adopts a helical structure, in which two 2,3′-bipyridyl units are twisted up and down relative to the plane of the central benzene ring. Weak inter­molecular C—H⋯π inter­actions lead to formation of a two-dimensional supra­molecular network. Hirshfeld surface analysis indicates that the mol­ecular packing in the title compound is mainly dominated by inter­molecular H⋯H and H⋯C/C⋯H inter­actions.

## Chemical context   

Phospho­rescent transition metal complexes based on platinum metal cations have attracted enormous current inter­est owing to their applications as electroluminescent devices, *e.g*. as phospho­rescent organic light-emitting diodes (PhOLEDs) or light-emitting electrochemical cells (LEECs) (Cebrián & Mauro, 2018 ▸). In particular, platinum complexes bearing tetra­dentate ligands are of great inter­est as blue phospho­rescent materials because of their pure blue emission and high efficiency (Fleetham *et al.*, 2014 ▸). It is well known that the origin of emission in platinum complexes results mainly from an intra-ligand charge transfer (ILCT) mixed with a metal-to-ligand charge-transfer transition (MLCT) (Yersin *et al.*, 2011 ▸). In order to achieve blue phospho­rescent materials, the design of ligands with a large triplet energy needs to be taken into account as the first step.

Our inter­est has been focused on the development of a suitable tetra­dentate ligand based on 2,3′-bi­pyridine with a large triplet energy (Lee *et al.*, 2017 ▸). Moreover, the crystal structures of 2,3′-bi­pyridine-based tetra­dentate ligands have aroused our curiosity, because the knowledge of the coordination mode(s) to a metal ion are of paramount importance in understanding its chemical and physical properties. Herein, we describe the mol­ecular and crystal structures of the title compound that can act as a tetra­dentate ligand to various transition metal ions. In addition, the mol­ecular packing of the title compound was examined with the aid of a Hirshfeld surface analysis.
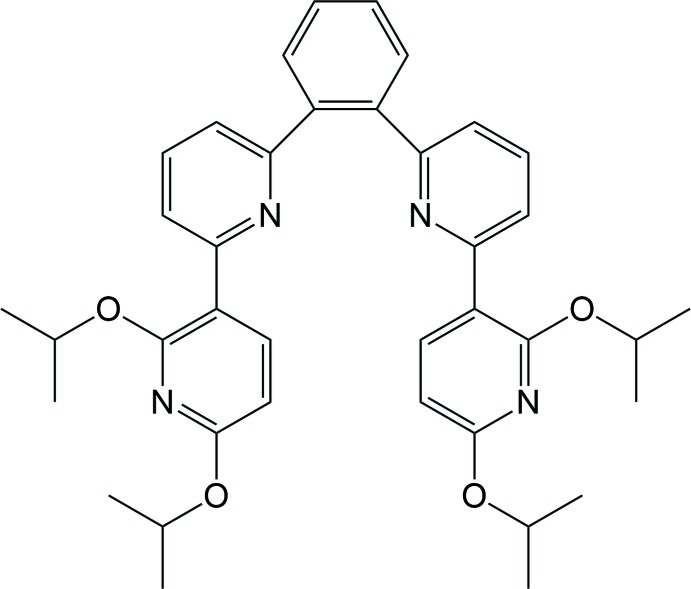


## Structural commentary   

**Figure 1:**
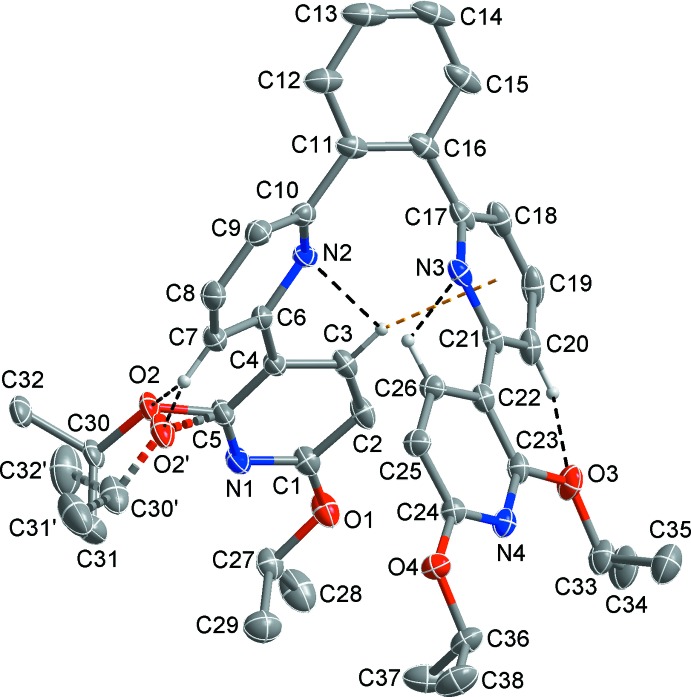
The mol­ecular structure of the title compound with the atom-numbering scheme. Displacement ellipsoids are drawn at the 30% probability level; H atoms not involved in intra­molecular inter­actions were omitted for clarity. The minor part of the disordered isopropyl group is drawn by two-coloured dashed lines. Black and yellow dashed lines represent intra­molecular C—H⋯N/O hydrogen bonds and C—H⋯π inter­actions.

The mol­ecular structure of the title compound is shown in Fig. 1 ▸. One isopropoxyl group is disordered over two sets of sites [C31–C30(–O2)–C32 and C31′–C30′(–O2′)–C32′, respectively]. Within the mol­ecule, intra­molecular C—H⋯N/O hydrogen bonds (Table 1 ▸, shown as black dashed lines in Fig. 1 ▸) are observed. With respect to the two 2,3′-bipyridyl units, the N1-containing pyridine ring is tilted by 31.78 (6)° relative to the attached N2-containing one, while the N3-containing pyridine ring is only slightly tilted by 11.89 (8)° to the attached N4-containing one. The central benzene ring linking to the two 2,3′-bipyridyl units is tilted by 39.84 (5) and 48.07 (5)° relative to N2- and N3-containing pyridine rings, respectively.

The two 2,3′-bipyridyl units are attached at the 1,2-positions of the central benzene in an up- and down-fashion with the C10—C11—C16—C17 torsion angle being −10.8 (2)°, which is believed to reduce the steric hindrance between the two 2,3′-bipyridyl units. In combination with this torsion angle, the consecutive connections of five aromatic rings in the title mol­ecule lead to a helical structure. The central benzene unit occupies *ortho*-positions relative to the N atoms (N2 and N3) of the two inner pyridine rings, while the outer pyridine rings containing N1 and N4 are substituted relative to the inner pyridine rings at the *meta*-positions. An intra­molecular C—H⋯π inter­action between aromatic H3 and the centroid of the N3/C17–C21 ring as well as C—H⋯N/O hydrogen bonds (Table 1 ▸, shown as yellow and black dashed lines in Fig. 1 ▸, respectively) assists in the stabilization of the helical structure.

## Supra­molecular features   

**Figure 2:**
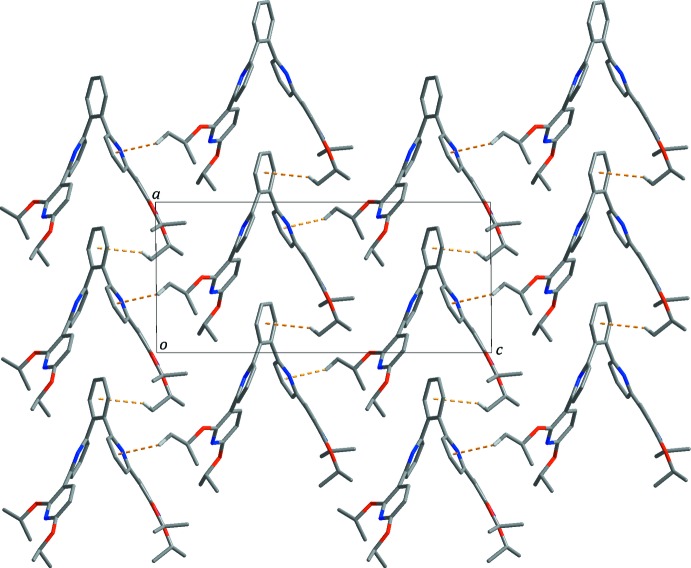
Layer formed through inter­molecular C—H⋯π inter­actions (yellow dashed lines). The disordered isopropoxyl group and H atoms not involved in inter­molecular inter­actions are not shown for clarity. Colour codes: grey = carbon, blue = nitro­gen, red = oxygen and white = hydrogen.

In the crystal structure, the title mol­ecules are inter­linked by further C—H⋯π inter­actions (Table 1 ▸, yellow dashed lines in Fig. 2 ▸) between (meth­yl)H32*A*⋯*Cg*1^i^ and between (meth­yl)H37*C*⋯*Cg*2^ii^ [*Cg*1 and *Cg*2 are the centroids of the N3/C17–C21 and C11–C16 rings, respectively; symmetry codes refer to Table 1 ▸], forming a two-dimensional supra­molecular network parallel to the *ac* plane, in which mol­ecules with right- and left-handed helical structures are alternately arranged. These layers are stacked in an *ABAB* fashion along the *b*-axis direction whereby no significant inter­molecular inter­actions between the layers are observed.

## Hirshfeld surface analysis   

**Figure 3:**
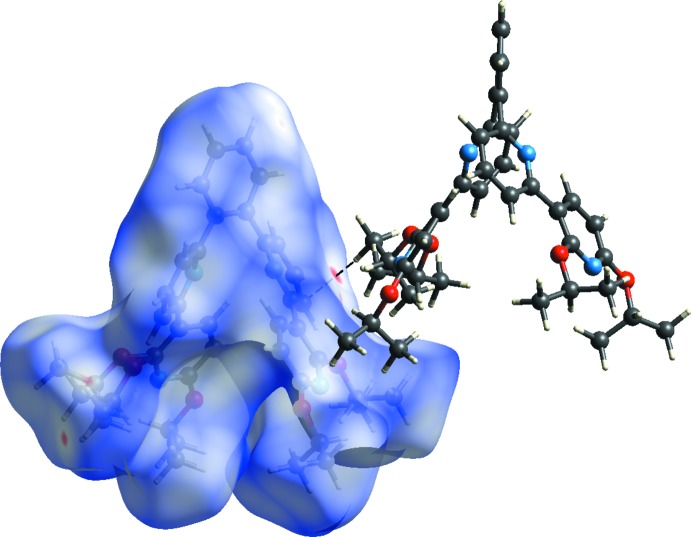
A view of the Hirshfeld surface of the title compound mapped over *d*_norm_, showing H⋯C contacts of inter­molecular C—H⋯π inter­actions using a fixed colour scale of −0.1511 (red) to 1.6184 (blue) a.u.

**Figure 4:**
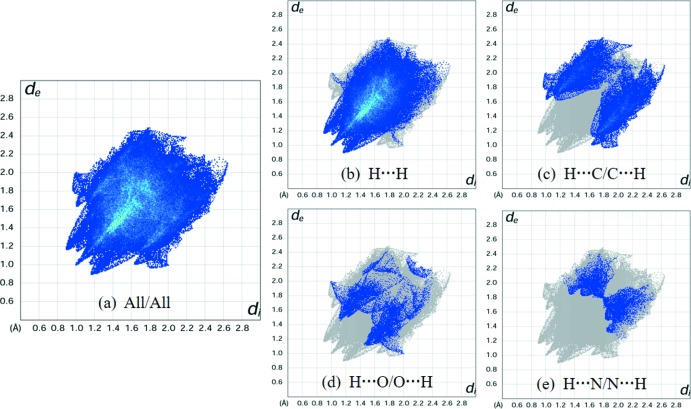
(*a*) The full two-dimensional fingerprint plot for the title compound and those delineated into (*b*) H⋯H, (*c*) H⋯C/C⋯H, (*d*) H⋯O/O⋯H and (*e*) H⋯N/N⋯H contacts. The *d*_i_ and *d*_e_ values are the closest inter­nal and external distances (in Å) from given points on the Hirshfeld surface contacts.

In order to qu­antify the various inter­molecular inter­actions in the mol­ecular packing of the title compound, a Hirshfeld surface analysis was carried out using *CrystalExplorer* (Turner *et al.*, 2017 ▸). In Fig. 3 ▸, which shows the Hirshfeld surface mapped over the normalized contact distance (*d*_norm_), the light-red spot on the surface indicates contact points with atoms participating in inter­molecular C—H⋯π inter­actions, corresponding to the H32*A* and pyridine-C20 atoms (Table 2 ▸). Except for this light-red spot, the overall surface mapped over *d*_norm_ is covered by white and blue colours, indicating that the distances between the contact atoms in inter­molecular contacts are nearly the same as the sum of their van der Waals radii or longer. Therefore, there are no effective inter­molecular inter­actions apart from the C—H⋯π inter­actions in the mol­ecular packing. These features are confirmed in the two-dimensional fingerprint plots, Fig. 4 ▸ *a*–*e*, delineated into overall, H⋯H, H⋯C/C⋯H, H⋯O/O⋯H and H⋯N/N⋯H contacts, respectively. Their relative contributions of inter­atomic contacts to the Hirshfeld surface are summarized in Table 3 ▸.

As shown in Fig. 4 ▸ *b* and Table 3 ▸, the most widely scattered points in the fingerprint plot are related to H⋯H contacts, which make a 65.2% contribution to the Hirshfeld surface. The sharp peak at *d*_e_ = *d*_i_ = 1.0 Å in the fingerprint plot delineated into H⋯H contacts (Fig. 4 ▸ *b*) corresponds to the shortest inter­atomic H⋯H contact between symmetry-related isoprop­oxy-H34*C* atoms (Table 2 ▸), whereas two pairs of the flanking broad peaks, symmetrically disposed with respect to the diagonal, at *d*_e_ + *d*_i_ ∼ 2.1 and 2.2 Å, result from inter­atomic H⋯H contacts between the isoprop­oxy-H34*B* and -H31*F* atoms and between the benzene-H18 and isoprop­oxy-H31*E* atoms, respectively (Table 2 ▸). The central green strip in Fig. 4 ▸ *b*, centered at *d*_e_ + *d*_i_ = 2.8 Å along the diagonal, indicates the presence of a large number of loose H⋯H contacts in the mol­ecular packing. The second largest contribution (22.7%) to the Hirshfeld surface of the title compound is due to inter­atomic H⋯C/C⋯H contacts (Fig. 4 ▸ *c* and Table 3 ▸), drawn on the fingerprint plot as a pair with a symmetrical wing-like shape on the left and right side with respect to the diagonal. The peaks at *d*_e_ + *d*_i_ ∼ 2.7 Å in the fingerprint plot delineated into H⋯C/C⋯H contacts (Fig. 4 ▸ *c*) reflect the presence of short C—H⋯π inter­actions between the isoprop­oxy-H32*A* and pyridine-C20 atoms (Table 2 ▸).

In the fingerprint plot delineated into H⋯O/O⋯H contacts (Fig. 4 ▸ *d*), the 6.5% contribution to the Hirshfeld surface (Table 3 ▸) originates from C—H⋯O hydrogen bonding. A pair of broad peaks at *d*_e_ + *d*_i_ ∼ 2.6 Å in Fig. 4 ▸ *d* corresponds to hydrogen bonding between the pyridine-H25 and O4 atoms (Table 2 ▸). Although N⋯H/H⋯N contacts with a contribution of 4.3% to the Hirshfeld surface (Fig. 4 ▸ *e* and Table 3 ▸) were observed, their inter­atomic distances are longer than the sum of their van der Waals radii and therefore they do not specifically contribute to the mol­ecular packing. Finally, the small contributions from the remaining inter­atomic contacts (Table 3 ▸), *i.e*. C⋯C (0.9%), N⋯C/C⋯N (0.4%) and O⋯C/C⋯O (0.1%), have a negligible effect on the mol­ecular packing.

In summary, the Hirshfeld surface analysis and two-dimensional fingerprint plot reveal that the mol­ecular packing in the title compound is dominated by inter­molecular van der Waals inter­actions between neighbouring H atoms as well as by C—H⋯π inter­actions.

## Database survey   

Although a search of the Cambridge Structural Database (CSD, Version 5.39, last update May 2018; Groom *et al.*, 2016 ▸) for 2′,6′-disubstituted 2,3′-bi­pyridine gave a number of hits, that for 2′,6′-dialk­oxy-2,3′-bi­pyridine gave only four hits. Three [FINJAP (Polander *et al.*, 2013 ▸), SITFIM (Frey *et al.*, 2014 ▸) and XIXNID (Oh *et al.*, 2013 ▸)] are Ru^II^ or Ir^II^ complexes with the ligand 2′,6′-dimeth­oxy-2,3′-bi­pyridine, and the remaining one (XIXNEZ; Oh *et al.*, 2013 ▸) is an Ir^II^ complex with the ligand 2′,6′-di(2-meth­oxy­eth­oxy-2,3′-bi­pyridine. Recently, our group has also reported the crystal structure of 2,3′-bi­pyridine-2′,6′-dicarbo­nitrile (Jung *et al.*, 2018 ▸) and the phospho­rescent properties for the Ir^II^ complex with ligand 2′,6′-diisoprop­oxy-2,3′-bi­pyridine (Kim *et al.*, 2018 ▸).

## Synthesis and crystallization   

**Figure 5:**
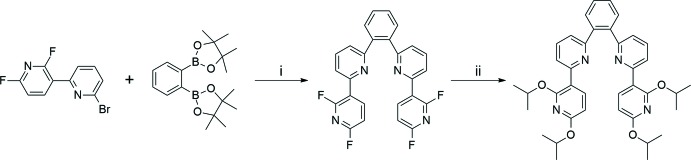
Synthetic routes and reagents to obtain the title compound: (i) 1,2-dipinacolato­benzene­(1.5 eq), Pd(PPh_3_)_4_ (6 mol%), 2 *M* K_3_PO_4_ (6 eq), THF, 363 K, 48 h; (ii) NaH (6 eq), ^*i*^PrOH (8 eq), DMF, 273 K, 10 h.

All experiments were performed under a dry N_2_ atmosphere using standard Schlenk techniques. All solvents were freshly distilled over appropriate drying reagents prior to use. All starting materials were commercially purchased and used without further purification. The ^1^H NMR spectrum was recorded on a Bruker Advance 400 MHz spectrometer. The two starting materials, 6-bromo-2′,6′-dii­fluoro-2,3′-bi­pyridine and 1,2-bis­(2′,6′-di­fluoro-2,3′-bi­pyridine)­benzene were synthesized according to a slight modification of the previous synthetic methodology reported by our group (Kim *et al.*, 2018 ▸; Oh *et al.*, 2013 ▸). Details regarding the synthetic procedures and reagents are presented in Fig. 5 ▸.

The title compound was synthesized as follows: NaH (0.063 g, 2.64 mmol) was dissolved in DMF (10 ml) at 273 K. Isopropyl alcohol (1.27 ml, 3.52 mmol) was added slowly at the same temperature. Then the reaction mixture was stirred for 30 min. 1,2-Bis(2′,6′-di­fluoro­bipyridine)­benzene (0.2 g, 0.44 mmol) in DMF (10 ml) was subsequently added into the reaction mixture, which was stirred at 273 K for a further 10 h. All volatiles were removed under vacuum and the remaining solid extracted with EtOAc. The pure title compound was obtained by silica column chromatography (EtOAc/hexane = 1/10 *v*/*v*). Colourless crystals with X-ray quality were obtained by slow evaporation of a di­chloro­methane solution of title compound. ^1^H NMR (400 MHz, CDCl_3_) δ 7.92 (*d*, *J* = 8.0 Hz, 2H), 7.75 (*dd*, *J* = 4.2 Hz, 2H), 7.64 (*t*, *J* = 8.0 Hz, 2H), 7.59 (*d*, *J* = 8.0 Hz, 2H), 7.53 (*dd*, *J* = 4.0 Hz, 2H), 7.22 (*d*, *J* = 7.7 Hz, 2H), 6.17 (*d*, *J* = 7.6 Hz, 2H), 5.38 (*sep*, *J* = 3.7 Hz, 2H), 5.23 (*sep*, *J* = 3.7 Hz, 2H) 1.40 (*d*, *J* = 6.5 Hz, 12H), 1.36 (*d*, *J* = 6.4 Hz, 12H).

## Refinement   

Crystal data, data collection and crystal structure refinement details are summarized in Table 4 ▸. All H atoms were positioned geometrically and refined using a riding model, with C—H = 0.95 Å for C*sp* ^2^—H, 1.00 Å for methine C—H, 0.98 Å for methyl C–H with *U*_iso_(H) = 1.2–1.5*U*_eq_(C). The isopropyl group [C31–C30(–O2)–C32] was found to be disordered over two sets of sites [occupancy ratio 0.715 (5):0.285 (5)].

## Supplementary Material

Crystal structure: contains datablock(s) I, New_Global_Publ_Block. DOI: 10.1107/S2056989018013002/wm5462sup1.cif

Structure factors: contains datablock(s) I. DOI: 10.1107/S2056989018013002/wm5462Isup2.hkl

CCDC reference: 1867774

Additional supporting information:  crystallographic information; 3D view; checkCIF report

## Figures and Tables

**Table 1:** Hydrogen-bond geometry (Å, °) *Cg*1 and *Cg*2 are the centroids of the N3/C17–C21 and C11–C16 rings, respectively.

*D*—H⋯*A*	*D*—H	H⋯*A*	*D*⋯*A*	*D*—H⋯*A*
C3—H3⋯N2	0.95	2.47	2.789 (2)	100
C7—H7⋯O2	0.95	2.50	2.985 (3)	111
C7—H7⋯O2′	0.95	2.07	2.694 (8)	122
C20—H20⋯O3	0.95	2.21	2.825 (2)	122
C26—H26⋯N3	0.95	2.37	2.726 (2)	102
C3—H3⋯*Cg*1	0.95	2.61	3.5078 (18)	158
C32—H32*A*⋯*Cg*1^i^	0.98	2.79	3.594 (3)	140
C37—H37*C*⋯*Cg*2^ii^	0.98	2.96	3.742 (3)	137

Symmetry codes: (i) 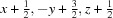; (ii) 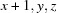.

**Table 2:** Summary of selected short inter­atomic contacts (Å) in the title compound

Contact	Distance	Symmetry operation
H34*C*⋯H34*C*	2.01	-*x* + 2, −*y* + 1, −*z*
H34*B*⋯H31*F*	2.08	-*x* + 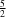 , *y* − 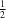 , −*z* + 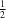
H18⋯H31*E*	2.14	-*x* + 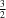 , *y* − 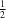 , −*z* + 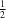
H32*A*⋯C20	2.66	*x* + 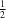 , −*y* + 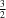 , *z* + 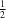
H25⋯O4	2.60	-*x* + 2, −*y* + 2, −*z*

**Table 3:** Percentage contributions of inter­atomic contacts to the Hirshfeld surface of the title compound

Contact	Percentage contribution
H⋯H	65.2
H⋯C/C⋯H	22.7
H⋯O/O⋯H	6.5
H⋯N/N⋯H	4.3
C⋯C	0.9
N⋯C/C⋯N	0.4
O⋯C/C⋯O	0.1

**Table 4:** Experimental details

Crystal data	
Chemical formula	C_38_H_42_N_4_O_4_
*M*_r_	618.75
Crystal system, space group	Monoclinic, *P*2_1_/*n*
Temperature (K)	173
*a*, *b*, *c* (Å)	9.4897 (2), 17.2533 (4), 21.0921 (5)
β (°)	90.4825 (13)
*V* (Å^3^)	3453.26 (14)
*Z*	4
Radiation type	Mo *K*α
μ (mm^−1^)	0.08
Crystal size (mm)	0.42 × 0.17 × 0.14

Data collection	
Diffractometer	Bruker APEXII CCD
Absorption correction	Multi-scan (*SADABS*; Bruker, 2014 ▸)
*T*_min_, *T*_max_	0.705, 0.746
No. of measured, independent and observed [*I* > 2σ(*I*)] reflections	55030, 6786, 5400
*R*_int_	0.042
(sin θ/λ)_max_ (Å^−1^)	0.617

Refinement	
*R*[*F* ^2^ > 2σ(*F* ^2^)], *wR*(*F* ^2^), *S*	0.048, 0.130, 1.05
No. of reflections	6786
No. of parameters	452
H-atom treatment	H-atom parameters constrained
Δρ_max_, Δρ_min_ (e Å^−3^)	0.18, −0.29

Computer programs: *APEX2* and *SAINT* (Bruker, 2014 ▸), *SHELXS97* and *SHELXTL* (Sheldrick, 2008 ▸), *SHELXL2014* (Sheldrick, 2015 ▸), *DIAMOND* (Brandenburg, 2010 ▸) and *publCIF* (Westrip, 2010 ▸).

## supplementary crystallographic information

### Crystal data

**Table:**

C_38_H_42_N_4_O_4_	*F*(000) = 1320
*M**_r_* = 618.75	*D*_x_ = 1.190 Mg m^−^^3^
Monoclinic, *P*2_1_/*n*	Mo *K*α radiation, λ = 0.71073 Å
*a* = 9.4897 (2) Å	Cell parameters from 9922 reflections
*b* = 17.2533 (4) Å	θ = 2.3–26.8°
*c* = 21.0921 (5) Å	µ = 0.08 mm^−^^1^
β = 90.4825 (13)°	*T* = 173 K
*V* = 3453.26 (14) Å^3^	Needle, colourless
*Z* = 4	0.42 × 0.17 × 0.14 mm

### Data collection

**Table:**

Bruker APEXII CCD diffractometer	5400 reflections with *I* > 2σ(*I*)
φ and ω scans	*R*_int_ = 0.042
Absorption correction: multi-scan (SADABS; Bruker, 2014)	θ_max_ = 26.0°, θ_min_ = 1.5°
*T*_min_ = 0.705, *T*_max_ = 0.746	*h* = −11→11
55030 measured reflections	*k* = −21→21
6786 independent reflections	*l* = −26→26

### Refinement

**Table:**

Refinement on *F*^2^	0 restraints
Least-squares matrix: full	Hydrogen site location: inferred from neighbouring sites
*R*[*F*^2^ > 2σ(*F*^2^)] = 0.048	H-atom parameters constrained
*wR*(*F*^2^) = 0.130	*w* = 1/[σ^2^(*F*_o_^2^) + (0.0499*P*)^2^ + 1.521*P*] where *P* = (*F*_o_^2^ + 2*F*_c_^2^)/3
*S* = 1.05	(Δ/σ)_max_ < 0.001
6786 reflections	Δρ_max_ = 0.18 e Å^−^^3^
452 parameters	Δρ_min_ = −0.29 e Å^−^^3^

### Special details

**Table:**

Geometry. All esds (except the esd in the dihedral angle between two l.s. planes) are estimated using the full covariance matrix. The cell esds are taken into account individually in the estimation of esds in distances, angles and torsion angles; correlations between esds in cell parameters are only used when they are defined by crystal symmetry. An approximate (isotropic) treatment of cell esds is used for estimating esds involving l.s. planes.

### Fractional atomic coordinates and isotropic or equivalent isotropic displacement parameters (Å^2^)

**Table:**

	*x*	*y*	*z*	*U*_iso_*/*U*_eq_	Occ. (<1)
O1	1.19196 (15)	0.58808 (7)	0.31447 (7)	0.0521 (4)	
O3	1.01870 (15)	0.65522 (7)	0.01321 (6)	0.0507 (3)	
O4	1.15126 (12)	0.90921 (7)	−0.01613 (6)	0.0411 (3)	
N1	1.09841 (15)	0.70868 (9)	0.33625 (7)	0.0428 (4)	
N2	0.66435 (14)	0.78701 (8)	0.23833 (6)	0.0336 (3)	
N3	0.64904 (15)	0.73193 (8)	0.10559 (6)	0.0359 (3)	
N4	1.08181 (15)	0.78198 (8)	−0.00226 (7)	0.0376 (3)	
C1	1.09115 (19)	0.64246 (10)	0.30500 (8)	0.0392 (4)	
C2	0.9834 (2)	0.62424 (10)	0.26277 (9)	0.0427 (4)	
H2	0.9794	0.5752	0.2423	0.051*	
C3	0.88276 (18)	0.67992 (10)	0.25170 (8)	0.0369 (4)	
H3	0.8066	0.6687	0.2237	0.044*	
C4	0.88968 (17)	0.75228 (10)	0.28048 (8)	0.0343 (4)	
C5	1.00019 (19)	0.76141 (11)	0.32417 (9)	0.0436 (4)	
C6	0.78887 (16)	0.81328 (10)	0.26010 (7)	0.0334 (4)	
C7	0.82307 (19)	0.89191 (10)	0.25820 (9)	0.0416 (4)	
H7	0.9107	0.9098	0.2747	0.050*	
C8	0.72757 (19)	0.94328 (11)	0.23194 (9)	0.0439 (4)	
H8	0.7490	0.9970	0.2300	0.053*	
C9	0.60074 (18)	0.91603 (10)	0.20854 (8)	0.0400 (4)	
H9	0.5341	0.9505	0.1900	0.048*	
C10	0.57258 (17)	0.83754 (10)	0.21263 (7)	0.0351 (4)	
C11	0.43609 (17)	0.80458 (11)	0.18974 (8)	0.0407 (4)	
C12	0.31225 (19)	0.84493 (14)	0.20206 (10)	0.0553 (5)	
H12	0.3168	0.8943	0.2218	0.066*	
C13	0.1815 (2)	0.81347 (18)	0.18565 (12)	0.0697 (7)	
H13	0.0979	0.8421	0.1934	0.084*	
C14	0.1732 (2)	0.74192 (18)	0.15866 (11)	0.0691 (8)	
H14	0.0837	0.7198	0.1492	0.083*	
C15	0.2946 (2)	0.70166 (14)	0.14506 (9)	0.0589 (6)	
H15	0.2878	0.6520	0.1258	0.071*	
C16	0.42795 (18)	0.73237 (11)	0.15907 (8)	0.0427 (4)	
C17	0.55456 (19)	0.68978 (11)	0.13724 (8)	0.0408 (4)	
C18	0.5718 (2)	0.61052 (11)	0.14788 (9)	0.0495 (5)	
H18	0.5020	0.5814	0.1694	0.059*	
C19	0.6925 (3)	0.57578 (11)	0.12631 (8)	0.0523 (5)	
H19	0.7071	0.5219	0.1331	0.063*	
C20	0.7932 (2)	0.61910 (10)	0.09469 (8)	0.0447 (4)	
H20	0.8776	0.5956	0.0802	0.054*	
C21	0.76775 (19)	0.69822 (9)	0.08465 (7)	0.0362 (4)	
C22	0.86729 (18)	0.75218 (9)	0.05339 (8)	0.0342 (4)	
C23	0.98940 (19)	0.73182 (9)	0.02104 (8)	0.0369 (4)	
C24	1.05673 (18)	0.85710 (9)	0.00537 (8)	0.0345 (4)	
C25	0.93616 (18)	0.88538 (10)	0.03383 (9)	0.0396 (4)	
H25	0.9183	0.9394	0.0366	0.048*	
C26	0.84346 (18)	0.83202 (10)	0.05790 (8)	0.0376 (4)	
H26	0.7606	0.8499	0.0782	0.045*	
C27	1.3110 (2)	0.60571 (11)	0.35629 (9)	0.0455 (4)	
H27	1.2762	0.6316	0.3956	0.055*	
C28	1.3729 (3)	0.52827 (13)	0.37302 (13)	0.0761 (8)	
H28A	1.4543	0.5356	0.4013	0.114*	
H28B	1.3018	0.4967	0.3944	0.114*	
H28C	1.4032	0.5019	0.3343	0.114*	
C29	1.4147 (2)	0.65754 (14)	0.32372 (12)	0.0654 (6)	
H29A	1.4937	0.6686	0.3526	0.098*	
H29B	1.4500	0.6317	0.2857	0.098*	
H29C	1.3683	0.7061	0.3117	0.098*	
O2	0.9987 (3)	0.82476 (17)	0.36266 (13)	0.0418 (6)	0.715 (5)
C30	1.1067 (3)	0.8352 (2)	0.41069 (19)	0.0411 (8)	0.715 (5)
H30	1.1288	0.7840	0.4307	0.049*	0.715 (5)
C31	1.2377 (4)	0.8682 (2)	0.3827 (2)	0.0655 (11)	0.715 (5)
H31A	1.3092	0.8747	0.4161	0.098*	0.715 (5)
H31B	1.2734	0.8330	0.3502	0.098*	0.715 (5)
H31C	1.2165	0.9187	0.3636	0.098*	0.715 (5)
C32	1.0413 (3)	0.88800 (17)	0.45934 (13)	0.0502 (9)	0.715 (5)
H32A	1.1093	0.8977	0.4937	0.075*	0.715 (5)
H32B	1.0153	0.9372	0.4393	0.075*	0.715 (5)
H32C	0.9569	0.8634	0.4767	0.075*	0.715 (5)
O2'	1.0329 (8)	0.8426 (4)	0.3350 (4)	0.054 (2)	0.285 (5)
C30'	1.1671 (13)	0.8612 (6)	0.3676 (5)	0.065 (3)	0.285 (5)
H30'	1.2446	0.8290	0.3495	0.078*	0.285 (5)
C31'	1.1936 (11)	0.9439 (5)	0.3539 (6)	0.093 (4)	0.285 (5)
H31D	1.2051	0.9510	0.3081	0.140*	0.285 (5)
H31E	1.1137	0.9750	0.3685	0.140*	0.285 (5)
H31F	1.2796	0.9606	0.3760	0.140*	0.285 (5)
C32'	1.1476 (19)	0.8413 (8)	0.4344 (6)	0.095 (5)	0.285 (5)
H32D	1.1308	0.7855	0.4383	0.142*	0.285 (5)
H32E	1.2324	0.8554	0.4586	0.142*	0.285 (5)
H32F	1.0665	0.8697	0.4510	0.142*	0.285 (5)
C33	1.1516 (2)	0.63360 (11)	−0.01576 (10)	0.0533 (5)	
H33	1.2264	0.6712	−0.0024	0.064*	
C34	1.1862 (3)	0.55423 (12)	0.01062 (12)	0.0717 (7)	
H34A	1.2754	0.5361	−0.0072	0.108*	
H34B	1.1948	0.5573	0.0569	0.108*	
H34C	1.1108	0.5179	−0.0008	0.108*	
C35	1.1371 (3)	0.63478 (15)	−0.08668 (11)	0.0717 (7)	
H35A	1.2270	0.6202	−0.1058	0.108*	
H35B	1.0639	0.5979	−0.0999	0.108*	
H35C	1.1108	0.6870	−0.1007	0.108*	
C36	1.2860 (2)	0.88172 (12)	−0.03986 (9)	0.0479 (5)	
H36	1.2707	0.8365	−0.0688	0.058*	
C37	1.3805 (2)	0.85865 (15)	0.01425 (13)	0.0716 (7)	
H37A	1.4707	0.8403	−0.0023	0.107*	
H37B	1.3968	0.9035	0.0419	0.107*	
H37C	1.3358	0.8171	0.0385	0.107*	
C38	1.3438 (3)	0.94945 (15)	−0.07685 (12)	0.0718 (7)	
H38A	1.4354	0.9354	−0.0946	0.108*	
H38B	1.2783	0.9627	−0.1114	0.108*	
H38C	1.3550	0.9942	−0.0486	0.108*	

### Atomic displacement parameters (Å^2^)

**Table:**

	*U*^11^	*U*^22^	*U*^33^	*U*^12^	*U*^13^	*U*^23^
O1	0.0617 (9)	0.0355 (7)	0.0586 (8)	0.0067 (6)	−0.0258 (7)	−0.0017 (6)
O3	0.0690 (9)	0.0287 (6)	0.0546 (8)	0.0051 (6)	0.0061 (7)	−0.0080 (6)
O4	0.0397 (7)	0.0347 (6)	0.0492 (7)	0.0022 (5)	0.0089 (5)	0.0048 (5)
N1	0.0384 (8)	0.0418 (9)	0.0482 (9)	0.0004 (6)	−0.0119 (7)	−0.0066 (7)
N2	0.0298 (7)	0.0424 (8)	0.0287 (7)	−0.0036 (6)	−0.0013 (5)	0.0013 (6)
N3	0.0406 (8)	0.0359 (8)	0.0311 (7)	−0.0090 (6)	−0.0090 (6)	0.0041 (6)
N4	0.0452 (8)	0.0330 (8)	0.0345 (7)	0.0049 (6)	−0.0007 (6)	−0.0019 (6)
C1	0.0455 (10)	0.0318 (9)	0.0401 (9)	−0.0018 (7)	−0.0077 (8)	0.0054 (7)
C2	0.0560 (11)	0.0272 (9)	0.0445 (10)	−0.0077 (8)	−0.0147 (8)	0.0024 (7)
C3	0.0431 (10)	0.0336 (9)	0.0339 (8)	−0.0100 (7)	−0.0094 (7)	0.0046 (7)
C4	0.0329 (8)	0.0372 (9)	0.0327 (8)	−0.0048 (7)	−0.0026 (7)	−0.0023 (7)
C5	0.0390 (10)	0.0399 (10)	0.0517 (11)	0.0008 (8)	−0.0117 (8)	−0.0128 (8)
C6	0.0318 (8)	0.0393 (9)	0.0292 (8)	−0.0023 (7)	−0.0008 (6)	−0.0056 (7)
C7	0.0373 (9)	0.0412 (10)	0.0463 (10)	−0.0036 (8)	−0.0054 (8)	−0.0100 (8)
C8	0.0483 (11)	0.0340 (9)	0.0493 (10)	−0.0012 (8)	0.0002 (8)	−0.0071 (8)
C9	0.0400 (10)	0.0420 (10)	0.0382 (9)	0.0052 (8)	0.0008 (7)	0.0005 (7)
C10	0.0319 (8)	0.0447 (10)	0.0286 (8)	−0.0008 (7)	0.0025 (6)	0.0022 (7)
C11	0.0308 (9)	0.0571 (11)	0.0343 (9)	−0.0055 (8)	−0.0022 (7)	0.0155 (8)
C12	0.0359 (10)	0.0785 (15)	0.0517 (12)	0.0033 (10)	0.0042 (8)	0.0211 (10)
C13	0.0307 (11)	0.112 (2)	0.0663 (15)	−0.0015 (12)	0.0019 (10)	0.0392 (15)
C14	0.0353 (11)	0.117 (2)	0.0549 (13)	−0.0264 (13)	−0.0136 (9)	0.0381 (14)
C15	0.0515 (12)	0.0828 (16)	0.0422 (11)	−0.0313 (11)	−0.0160 (9)	0.0246 (10)
C16	0.0399 (10)	0.0559 (11)	0.0322 (9)	−0.0160 (8)	−0.0094 (7)	0.0166 (8)
C17	0.0493 (10)	0.0432 (10)	0.0297 (8)	−0.0177 (8)	−0.0124 (7)	0.0046 (7)
C18	0.0732 (14)	0.0402 (10)	0.0349 (9)	−0.0224 (10)	−0.0064 (9)	0.0041 (8)
C19	0.0938 (16)	0.0299 (9)	0.0332 (9)	−0.0129 (10)	−0.0073 (10)	0.0008 (7)
C20	0.0714 (13)	0.0302 (9)	0.0322 (9)	−0.0039 (8)	−0.0069 (8)	−0.0031 (7)
C21	0.0502 (10)	0.0314 (9)	0.0268 (8)	−0.0066 (7)	−0.0113 (7)	−0.0004 (6)
C22	0.0423 (9)	0.0285 (8)	0.0315 (8)	−0.0022 (7)	−0.0075 (7)	0.0003 (6)
C23	0.0506 (10)	0.0266 (8)	0.0334 (9)	0.0026 (7)	−0.0070 (7)	−0.0029 (7)
C24	0.0389 (9)	0.0315 (9)	0.0331 (8)	0.0007 (7)	−0.0026 (7)	0.0012 (7)
C25	0.0400 (9)	0.0268 (8)	0.0522 (11)	0.0022 (7)	0.0028 (8)	−0.0006 (7)
C26	0.0362 (9)	0.0317 (9)	0.0449 (10)	0.0006 (7)	0.0004 (7)	−0.0004 (7)
C27	0.0530 (11)	0.0430 (10)	0.0402 (10)	0.0040 (8)	−0.0147 (8)	0.0031 (8)
C28	0.0897 (18)	0.0510 (13)	0.0869 (18)	0.0104 (12)	−0.0427 (15)	0.0095 (12)
C29	0.0568 (13)	0.0744 (16)	0.0651 (14)	0.0037 (11)	−0.0008 (11)	0.0119 (12)
O2	0.0425 (13)	0.0432 (14)	0.0393 (14)	0.0052 (10)	−0.0186 (11)	−0.0134 (11)
C30	0.0413 (17)	0.0456 (16)	0.036 (2)	0.0002 (13)	−0.0191 (15)	−0.0097 (16)
C31	0.047 (2)	0.080 (3)	0.070 (3)	−0.011 (2)	−0.0051 (18)	−0.019 (2)
C32	0.0513 (17)	0.0576 (18)	0.0413 (15)	0.0041 (13)	−0.0177 (12)	−0.0161 (13)
O2'	0.055 (4)	0.049 (4)	0.058 (5)	0.015 (3)	−0.030 (4)	−0.019 (3)
C30'	0.066 (7)	0.053 (5)	0.076 (7)	0.022 (5)	−0.035 (6)	−0.016 (5)
C31'	0.091 (7)	0.060 (6)	0.128 (9)	0.005 (5)	−0.049 (6)	−0.026 (6)
C32'	0.139 (14)	0.090 (8)	0.055 (8)	0.016 (8)	−0.034 (7)	−0.008 (6)
C33	0.0663 (13)	0.0396 (10)	0.0538 (12)	0.0129 (9)	−0.0023 (10)	−0.0108 (9)
C34	0.1011 (19)	0.0387 (12)	0.0753 (16)	0.0180 (12)	−0.0054 (14)	−0.0117 (11)
C35	0.0918 (18)	0.0706 (16)	0.0530 (13)	0.0188 (13)	0.0038 (12)	−0.0097 (11)
C36	0.0468 (11)	0.0470 (11)	0.0502 (11)	0.0078 (8)	0.0165 (9)	0.0063 (9)
C37	0.0435 (12)	0.0834 (17)	0.0880 (18)	0.0039 (11)	0.0008 (11)	0.0283 (14)
C38	0.0688 (15)	0.0722 (16)	0.0751 (16)	0.0081 (12)	0.0332 (13)	0.0229 (13)

### Geometric parameters (Å, º)

**Table:**

O1—C1	1.354 (2)	C24—C25	1.385 (2)
O1—C27	1.459 (2)	C25—C26	1.374 (2)
O3—C23	1.361 (2)	C25—H25	0.9500
O3—C33	1.455 (2)	C26—H26	0.9500
O4—C24	1.351 (2)	C27—C28	1.501 (3)
O4—C36	1.457 (2)	C27—C29	1.501 (3)
N1—C1	1.321 (2)	C27—H27	1.0000
N1—C5	1.326 (2)	C28—H28A	0.9800
N2—C6	1.343 (2)	C28—H28B	0.9800
N2—C10	1.343 (2)	C28—H28C	0.9800
N3—C17	1.337 (2)	C29—H29A	0.9800
N3—C21	1.346 (2)	C29—H29B	0.9800
N4—C24	1.328 (2)	C29—H29C	0.9800
N4—C23	1.329 (2)	O2—C30	1.446 (4)
C1—C2	1.386 (2)	C30—C31	1.494 (6)
C2—C3	1.373 (2)	C30—C32	1.510 (5)
C2—H2	0.9500	C30—H30	1.0000
C3—C4	1.390 (2)	C31—H31A	0.9800
C3—H3	0.9500	C31—H31B	0.9800
C4—C5	1.399 (2)	C31—H31C	0.9800
C4—C6	1.484 (2)	C32—H32A	0.9800
C5—O2	1.362 (3)	C32—H32B	0.9800
C5—O2'	1.453 (8)	C32—H32C	0.9800
C6—C7	1.396 (2)	O2'—C30'	1.478 (14)
C7—C8	1.380 (3)	C30'—C32'	1.464 (17)
C7—H7	0.9500	C30'—C31'	1.478 (15)
C8—C9	1.380 (2)	C30'—H30'	1.0000
C8—H8	0.9500	C31'—H31D	0.9800
C9—C10	1.383 (2)	C31'—H31E	0.9800
C9—H9	0.9500	C31'—H31F	0.9800
C10—C11	1.491 (2)	C32'—H32D	0.9800
C11—C12	1.392 (3)	C32'—H32E	0.9800
C11—C16	1.406 (3)	C32'—H32F	0.9800
C12—C13	1.395 (3)	C33—C35	1.501 (3)
C12—H12	0.9500	C33—C34	1.513 (3)
C13—C14	1.362 (4)	C33—H33	1.0000
C13—H13	0.9500	C34—H34A	0.9800
C14—C15	1.378 (4)	C34—H34B	0.9800
C14—H14	0.9500	C34—H34C	0.9800
C15—C16	1.401 (2)	C35—H35A	0.9800
C15—H15	0.9500	C35—H35B	0.9800
C16—C17	1.485 (3)	C35—H35C	0.9800
C17—C18	1.395 (3)	C36—C37	1.499 (3)
C18—C19	1.374 (3)	C36—C38	1.510 (3)
C18—H18	0.9500	C36—H36	1.0000
C19—C20	1.389 (3)	C37—H37A	0.9800
C19—H19	0.9500	C37—H37B	0.9800
C20—C21	1.402 (2)	C37—H37C	0.9800
C20—H20	0.9500	C38—H38A	0.9800
C21—C22	1.485 (2)	C38—H38B	0.9800
C22—C23	1.395 (2)	C38—H38C	0.9800
C22—C26	1.399 (2)		
			
C1—O1—C27	119.15 (14)	O1—C27—H27	109.6
C23—O3—C33	118.60 (15)	C28—C27—H27	109.6
C24—O4—C36	119.07 (13)	C29—C27—H27	109.6
C1—N1—C5	117.66 (15)	C27—C28—H28A	109.5
C6—N2—C10	118.98 (15)	C27—C28—H28B	109.5
C17—N3—C21	119.70 (15)	H28A—C28—H28B	109.5
C24—N4—C23	118.12 (15)	C27—C28—H28C	109.5
N1—C1—O1	119.49 (15)	H28A—C28—H28C	109.5
N1—C1—C2	123.50 (16)	H28B—C28—H28C	109.5
O1—C1—C2	117.00 (16)	C27—C29—H29A	109.5
C3—C2—C1	117.30 (16)	C27—C29—H29B	109.5
C3—C2—H2	121.3	H29A—C29—H29B	109.5
C1—C2—H2	121.3	C27—C29—H29C	109.5
C2—C3—C4	121.60 (15)	H29A—C29—H29C	109.5
C2—C3—H3	119.2	H29B—C29—H29C	109.5
C4—C3—H3	119.2	C5—O2—C30	120.4 (3)
C3—C4—C5	114.92 (15)	O2—C30—C31	111.0 (4)
C3—C4—C6	118.87 (14)	O2—C30—C32	105.0 (2)
C5—C4—C6	126.08 (15)	C31—C30—C32	112.7 (3)
N1—C5—O2	116.54 (18)	O2—C30—H30	109.4
N1—C5—C4	124.84 (16)	C31—C30—H30	109.4
O2—C5—C4	118.15 (18)	C32—C30—H30	109.4
N1—C5—O2'	118.9 (3)	C30—C31—H31A	109.5
C4—C5—O2'	111.7 (3)	C30—C31—H31B	109.5
N2—C6—C7	121.50 (15)	H31A—C31—H31B	109.5
N2—C6—C4	115.02 (14)	C30—C31—H31C	109.5
C7—C6—C4	123.26 (15)	H31A—C31—H31C	109.5
C8—C7—C6	118.93 (16)	H31B—C31—H31C	109.5
C8—C7—H7	120.5	C30—C32—H32A	109.5
C6—C7—H7	120.5	C30—C32—H32B	109.5
C9—C8—C7	119.52 (17)	H32A—C32—H32B	109.5
C9—C8—H8	120.2	C30—C32—H32C	109.5
C7—C8—H8	120.2	H32A—C32—H32C	109.5
C8—C9—C10	118.67 (16)	H32B—C32—H32C	109.5
C8—C9—H9	120.7	C5—O2'—C30'	117.6 (6)
C10—C9—H9	120.7	C32'—C30'—C31'	115.9 (10)
N2—C10—C9	122.39 (15)	C32'—C30'—O2'	106.4 (14)
N2—C10—C11	116.20 (15)	C31'—C30'—O2'	105.4 (7)
C9—C10—C11	121.39 (16)	C32'—C30'—H30'	109.6
C12—C11—C16	119.07 (17)	C31'—C30'—H30'	109.6
C12—C11—C10	118.77 (18)	O2'—C30'—H30'	109.6
C16—C11—C10	122.08 (16)	C30'—C31'—H31D	109.5
C11—C12—C13	120.6 (2)	C30'—C31'—H31E	109.5
C11—C12—H12	119.7	H31D—C31'—H31E	109.5
C13—C12—H12	119.7	C30'—C31'—H31F	109.5
C14—C13—C12	120.3 (2)	H31D—C31'—H31F	109.5
C14—C13—H13	119.8	H31E—C31'—H31F	109.5
C12—C13—H13	119.8	C30'—C32'—H32D	109.5
C13—C14—C15	119.9 (2)	C30'—C32'—H32E	109.5
C13—C14—H14	120.1	H32D—C32'—H32E	109.5
C15—C14—H14	120.1	C30'—C32'—H32F	109.5
C14—C15—C16	121.4 (2)	H32D—C32'—H32F	109.5
C14—C15—H15	119.3	H32E—C32'—H32F	109.5
C16—C15—H15	119.3	O3—C33—C35	110.02 (18)
C15—C16—C11	118.57 (19)	O3—C33—C34	105.29 (18)
C15—C16—C17	118.63 (19)	C35—C33—C34	113.36 (18)
C11—C16—C17	122.67 (15)	O3—C33—H33	109.3
N3—C17—C18	122.36 (19)	C35—C33—H33	109.3
N3—C17—C16	115.68 (16)	C34—C33—H33	109.3
C18—C17—C16	121.94 (17)	C33—C34—H34A	109.5
C19—C18—C17	118.11 (18)	C33—C34—H34B	109.5
C19—C18—H18	120.9	H34A—C34—H34B	109.5
C17—C18—H18	120.9	C33—C34—H34C	109.5
C18—C19—C20	120.24 (18)	H34A—C34—H34C	109.5
C18—C19—H19	119.9	H34B—C34—H34C	109.5
C20—C19—H19	119.9	C33—C35—H35A	109.5
C19—C20—C21	118.55 (19)	C33—C35—H35B	109.5
C19—C20—H20	120.7	H35A—C35—H35B	109.5
C21—C20—H20	120.7	C33—C35—H35C	109.5
N3—C21—C20	121.01 (16)	H35A—C35—H35C	109.5
N3—C21—C22	114.35 (14)	H35B—C35—H35C	109.5
C20—C21—C22	124.60 (17)	O4—C36—C37	110.25 (17)
C23—C22—C26	114.63 (15)	O4—C36—C38	104.41 (15)
C23—C22—C21	126.42 (15)	C37—C36—C38	112.41 (19)
C26—C22—C21	118.89 (16)	O4—C36—H36	109.9
N4—C23—O3	116.84 (16)	C37—C36—H36	109.9
N4—C23—C22	124.77 (15)	C38—C36—H36	109.9
O3—C23—C22	118.39 (15)	C36—C37—H37A	109.5
N4—C24—O4	119.25 (15)	C36—C37—H37B	109.5
N4—C24—C25	123.08 (16)	H37A—C37—H37B	109.5
O4—C24—C25	117.66 (15)	C36—C37—H37C	109.5
C26—C25—C24	117.26 (16)	H37A—C37—H37C	109.5
C26—C25—H25	121.4	H37B—C37—H37C	109.5
C24—C25—H25	121.4	C36—C38—H38A	109.5
C25—C26—C22	122.02 (16)	C36—C38—H38B	109.5
C25—C26—H26	119.0	H38A—C38—H38B	109.5
C22—C26—H26	119.0	C36—C38—H38C	109.5
O1—C27—C28	104.85 (16)	H38A—C38—H38C	109.5
O1—C27—C29	110.78 (16)	H38B—C38—H38C	109.5
C28—C27—C29	112.4 (2)		
			
C5—N1—C1—O1	177.89 (17)	C21—N3—C17—C16	179.28 (14)
C5—N1—C1—C2	−3.0 (3)	C15—C16—C17—N3	129.29 (17)
C27—O1—C1—N1	−3.5 (3)	C11—C16—C17—N3	−46.7 (2)
C27—O1—C1—C2	177.30 (16)	C15—C16—C17—C18	−49.3 (2)
N1—C1—C2—C3	2.5 (3)	C11—C16—C17—C18	134.79 (18)
O1—C1—C2—C3	−178.35 (16)	N3—C17—C18—C19	1.8 (3)
C1—C2—C3—C4	1.3 (3)	C16—C17—C18—C19	−179.71 (16)
C2—C3—C4—C5	−4.2 (3)	C17—C18—C19—C20	−0.3 (3)
C2—C3—C4—C6	171.91 (16)	C18—C19—C20—C21	−0.9 (3)
C1—N1—C5—O2	171.6 (2)	C17—N3—C21—C20	0.9 (2)
C1—N1—C5—C4	−0.4 (3)	C17—N3—C21—C22	−176.82 (14)
C1—N1—C5—O2'	−154.3 (5)	C19—C20—C21—N3	0.6 (2)
C3—C4—C5—N1	3.8 (3)	C19—C20—C21—C22	178.09 (15)
C6—C4—C5—N1	−171.94 (17)	N3—C21—C22—C23	−172.01 (15)
C3—C4—C5—O2	−168.0 (2)	C20—C21—C22—C23	10.3 (3)
C6—C4—C5—O2	16.2 (3)	N3—C21—C22—C26	10.9 (2)
C3—C4—C5—O2'	159.4 (4)	C20—C21—C22—C26	−166.77 (16)
C6—C4—C5—O2'	−16.4 (5)	C24—N4—C23—O3	−179.21 (15)
C10—N2—C6—C7	1.8 (2)	C24—N4—C23—C22	−0.3 (2)
C10—N2—C6—C4	−172.99 (14)	C33—O3—C23—N4	4.1 (2)
C3—C4—C6—N2	28.8 (2)	C33—O3—C23—C22	−174.92 (15)
C5—C4—C6—N2	−155.54 (17)	C26—C22—C23—N4	2.6 (2)
C3—C4—C6—C7	−145.87 (17)	C21—C22—C23—N4	−174.62 (15)
C5—C4—C6—C7	29.7 (3)	C26—C22—C23—O3	−178.47 (15)
N2—C6—C7—C8	−1.5 (3)	C21—C22—C23—O3	4.3 (3)
C4—C6—C7—C8	172.86 (16)	C23—N4—C24—O4	177.95 (14)
C6—C7—C8—C9	0.3 (3)	C23—N4—C24—C25	−3.0 (3)
C7—C8—C9—C10	0.5 (3)	C36—O4—C24—N4	−7.6 (2)
C6—N2—C10—C9	−0.9 (2)	C36—O4—C24—C25	173.24 (16)
C6—N2—C10—C11	−179.67 (14)	N4—C24—C25—C26	3.6 (3)
C8—C9—C10—N2	−0.2 (3)	O4—C24—C25—C26	−177.36 (15)
C8—C9—C10—C11	178.43 (16)	C24—C25—C26—C22	−1.0 (3)
N2—C10—C11—C12	137.74 (17)	C23—C22—C26—C25	−1.9 (2)
C9—C10—C11—C12	−41.0 (2)	C21—C22—C26—C25	175.57 (16)
N2—C10—C11—C16	−39.0 (2)	C1—O1—C27—C28	161.82 (19)
C9—C10—C11—C16	142.30 (17)	C1—O1—C27—C29	−76.7 (2)
C16—C11—C12—C13	1.5 (3)	N1—C5—O2—C30	4.5 (4)
C10—C11—C12—C13	−175.26 (17)	C4—C5—O2—C30	177.1 (3)
C11—C12—C13—C14	1.6 (3)	C5—O2—C30—C31	81.2 (4)
C12—C13—C14—C15	−2.7 (3)	C5—O2—C30—C32	−156.7 (3)
C13—C14—C15—C16	0.7 (3)	N1—C5—O2'—C30'	−8.6 (10)
C14—C15—C16—C11	2.3 (3)	C4—C5—O2'—C30'	−165.8 (8)
C14—C15—C16—C17	−173.77 (17)	C5—O2'—C30'—C32'	−74.0 (11)
C12—C11—C16—C15	−3.4 (2)	C5—O2'—C30'—C31'	162.4 (8)
C10—C11—C16—C15	173.27 (15)	C23—O3—C33—C35	−84.9 (2)
C12—C11—C16—C17	172.54 (16)	C23—O3—C33—C34	152.61 (17)
C10—C11—C16—C17	−10.8 (2)	C24—O4—C36—C37	−75.5 (2)
C21—N3—C17—C18	−2.2 (2)	C24—O4—C36—C38	163.58 (17)

### Hydrogen-bond geometry (Å, º)

*Cg*1 and *Cg*2 are the centroids of the N3/C17–C21 and C11–C16 rings, respectively.

**Table:**

*D*—H···*A*	*D*—H	H···*A*	*D*···*A*	*D*—H···*A*
C3—H3···N2	0.95	2.47	2.789 (2)	100
C7—H7···O2	0.95	2.50	2.985 (3)	111
C7—H7···O2′	0.95	2.07	2.694 (8)	122
C20—H20···O3	0.95	2.21	2.825 (2)	122
C26—H26···N3	0.95	2.37	2.726 (2)	102
C3—H3···*Cg*1	0.95	2.61	3.5078 (18)	158
C32—H32*A*···*Cg*1^i^	0.98	2.79	3.594 (3)	140
C37—H37*C*···*Cg*2^ii^	0.98	2.96	3.742 (3)	137

Symmetry codes: (i) *x*+1/2, −*y*+3/2, *z*+1/2; (ii) *x*+1, *y*, *z*.
